# Integrative analysis of bulk and single-cell gene expression profiles to identify bone marrow mesenchymal cell heterogeneity and prognostic significance in multiple myeloma

**DOI:** 10.1186/s12967-025-06637-6

**Published:** 2025-06-16

**Authors:** Fei-Er Ju, Bei-Hui Huang, Hao Wu, Bo Zou, Shu-Na Chen, Xiao-Yan Sang, Wei-Yao Liang, Zi-Xuan Liu, Zi-Xuan Zhang, Zi-Yi Yang, Yan-Ting Liang, Yue-Lan Liang, Huan Liu, Zhao-Xia Dong, Xue-Qi Liu, Li-Yuan Zheng, Jin-Cheng Zeng, Jin-Heng Wang, Lin Qi, Xing-Ding Zhang, Yongjiang Zheng, Juan Li

**Affiliations:** 1 Department of Hematology, The First Affiliated Hospital of Sun Yat-Sen University, Guangzhou, China; 2 Shenzhen Key Laboratory for Systems Medicine in Inflammatory Diseases, School of Medicine, Shenzhen Campus of Sun Yat-Sen University, Sun Yat-Sen University, Shenzhen, China; 3 Department of Hematology, Institute of Hematology, The Third Affiliated Hospital of Sun Yat-Sen University, Guangzhou, China; 4 Dongguan Key Laboratory of Medical Bioactive Molecular Developmental and Translational Research, Guangdong Provincial Key Laboratory of Medical Immunology and Molecular Diagnostics, Guangdong Medical University, Dongguan, China; 5 Xinghai Institute of Cell, Guangdong Xianhua Institute for Medical Research, Dongguan, China; 6 Guangzhou Municipal and Guangdong Provincial Key Laboratory of Protein Modification and Degradation, School of Basic Medical Sciences, Guangzhou Medical University, Guangzhou, China; 7 Cancer Research Center, School of Medicine, Xiamen University, Xiamen, 361102 China

**Keywords:** Multiple myeloma, Mesenchymal stem cells, Single-cell RNA sequencing, Immunosuppressive microenvironment, High mobility group proteins, Osteogenesis, Prognostic model

## Abstract

**Background:**

Multiple myeloma is a hematologic malignancy characterized by complex interactions within the tumor microenvironment, where mesenchymal stem cells (MSCs) contribute significantly to disease progression, immune suppression, and drug resistance.

**Methods:**

This study investigated the heterogeneity of MSCs in multiple myeloma using single-cell RNA sequencing (10X) and bulk transcriptomic data. Further analysis was performed by Seurat, SCENIC, CellChat. GSE4581 and GSE136337 were used as training set and validation set to construct a newly described prognostic model through COX and LASSO.

**Results:**

By analyzing bone marrow samples from healthy donors and multiple myeloma patients at different Revised International Staging System (R-ISS) stages, this study identified distinct MSC subpopulations, including osteogenic, angiogenic, immune regulatory, and multipotent clusters, each of which plays unique roles in the tumor microenvironment. Interestingly, we found a unique subclone with upregulated expression of high mobility group proteins, these MSC exert a strong regulatory effect, which was defined as “HMGhMSC”.

**Conclusions:**

Our findings reveal that the proportion of osteogenic MSCs, which are crucial for bone health, decreases as the disease progresses, which is correlated with the bone lysis commonly observed in advanced multiple myeloma. Additionally, immune regulatory MSCs contribute to the formation of an immunosuppressive microenvironment, promoting tumor immune evasion. A prognostic model based on HMGhMSC subpopulations was developed, which demonstrated that these cells have significant potential as therapeutic targets for improving the prognosis and developing treatments for bone disease in multiple myeloma patients.

**Supplementary Information:**

The online version contains supplementary material available at 10.1186/s12967-025-06637-6.

## Background

Multiple myeloma is a difficult-to-cure hematological malignancy characterized by abnormal proliferation of plasma cells that inevitably leads to chemotherapy resistance, disease relapse, and poor prognosis. Despite many new therapeutic targets and regimens being researched, extensive tumor heterogeneity results in significant variability in the response of multiple myeloma (MM) patients to treatment, making precision medicine a major challenge for MM patients.

Recent single-cell studies have characterized the inherent subclonal heterogeneity and longitudinal evolution of MM tumors, further emphasizing the need for a detailed understanding of myeloma tumor epitopes [1]. In fact, subclone-specific microenvironmental impacts and drug responses in refractory multiple myeloma have been revealed by single-cell transcriptomics [1–8]. In relapsed and refractory multiple myeloma (RRMM), Tirier et al. reported that RRMM cells possess unique mutations in 1q + and that they shape an immunosuppressive bone marrow environment (BME) by upregulating inflammatory cytokines and closely interacting with the bone marrow niche, characterized by the accumulation of PD1 + γδ T cells and tumor-associated macrophages, as well as the depletion of hematopoietic progenitor cells [9]. Genetic heterogeneity exists at all stages of the disease, from monoclonal gammopathy of undetermined significance and smoldering MM to MM. Dang et al. compared patients with precursor myeloma to those with myeloma and normal donors, revealing early genomic drivers of malignant transformation, such as ASS1, CCND2, and KLF2. They identified different evolutionary patterns and early immunological changes from precursor myeloma to myeloma [10].

Previous studies have confirmed that MM cells induce immune suppression, including receptor signaling dysregulation; cytokine expression; the expansion of regulatory T cells (Tregs), myeloid-derived suppressor cells (MDSCs), and tumor-associated macrophages; and NK cell dysfunction [11–13]. Changes in the microenvironment are associated with reduced antitumor responses, induced angiogenesis, chemotherapy resistance, and disease progression. Zavidij et al. used single-cell transcriptomics to analyze the CD138 + or CD45 + cell components in the bone marrow of MGUS, SMM, and overt MM patients and reported that the tumor microenvironment began to significantly change from the MGUS stage, with substantial enrichment of NK cells, T cells, and nonclassical monocytes and macrophages [14]. In addition, the presence of mesenchymal stem cells (MSCs) and cancer-associated fibroblasts (CAFs), which significantly influence tumor progression, in the TME has attracted increasing attention. Changes in the stroma in multiple myeloma patients have been reported to be associated with complications, including hematopoietic dysfunction and bone lysis [15]. In fact, studies have shown that some unique subpopulations of mesenchymal stem cells in multiple myeloma highly express the Toll-like receptor 4 (TLR4) gene, which in turn increases the expression of CD54 and interleukin-6 (IL-6), which is directly related to the crosstalk between MM MSCs and MM cells [16]. Compared with healthy donors, MM-MSCs express higher levels of IL-8, which enhances NF-κB activity in MM cells, leading to resistance to bortezomib. In addition, messenger RNA sequencing revealed underlying molecular signatures in different disease stages from MGUS to MM. During progression, BM-MSCs exhibit cytogenetic abnormalities, changes in differentiation tendency, and alterations in related gene expression levels [17].

However, there has not yet been an in-depth discussion about the role of MSCs in multiple myeloma at the single-cell level. Here, we processed samples from healthy donors and MM patients at different R-ISS stages for single-cell transcriptome sequencing. Our work revealed significant heterogeneity in MM-MSCs, revealing a unique genetic landscape. HMGhMSCs can regulate an immunosuppressive microenvironment through extracellular matrix remodeling, cytokine secretion, or direct cell contact, especially in R-ISS I. We also observed a gradual decrease in the osteogenic differentiation propensity of MSCs with R-ISS progression, indicating potential therapeutic candidates for osteolysis in multiple myeloma. This study conducted a comprehensive analysis of scRNA-seq data and chip data, revealing the heterogeneous gene landscape of human MM-related MSCs, providing possibilities for prognostic analysis and therapeutic improvement in MM.

## Methods

### Patient samples and cell preparation

We studied four patients with newly diagnosed multiple myeloma (NDMM). All patients provided written informed consent before participating in the study. The research was approved by the Ethics Committee of Sun Yat-sen University School of Medicine. Bone marrow aspirates were first centrifuged at 3000 rpm for 10 min to remove the upper layer of pale yellow transparent serum. The remaining fluid was diluted in a 1:1 ratio with the prepared buffer (PBS containing 0.1% BSA and 2 mM EDTA) and mononuclear cells were isolated by density centrifugation (Ficoll separation solution, Absin). The diluted bone marrow cells were centrifuged at 1800 rpm for 40 min, with an acceleration of 2 and a deceleration of 0. The cells were carefully aspirated and washed with the prepared buffer (centrifuged at 1800 rpm for 10 min). A 10 × red blood cell lysis buffer (Solarbio) was used to lyse RBCs at room temperature for 5 min, after which the bone marrow cells were washed twice (centrifuged at 500 g for 5 min) and resuspended in the prepared buffer. After cell counting, 1 × 10^7^ cells were separated by magnetic-activated cell sorting (using anti-CD138 microbeads, Miltenyi Biotec) according to the manufacturer’s protocol. Subsequently, up to 2.5 × 10^6^ CD138 negative cells were frozen in 90% FCS (Corning) and 10% DMSO (Sigma-Aldrich) and stored in liquid nitrogen for future use.

### Single-cell RNA library preparation and sequencing

Cellular suspensions were loaded on a 10X Genomics GemCode Single-cell instrument that generates single-cell Gel Bead-In-EMlusion (GEMs). Libraries were generated and sequenced from the cDNAs with Chromium Next GEM Single Cell 3’ Reagent Kits v3.1. Upon dissolution of the Gel Bead in a GEM, primers containing (i) an Illumina^®^ R1 sequence (read 1 sequencing primer), (ii) a 16 nt 10 × Barcode, (iii) a 10 nt Unique Molecular Identifier (UMI), and (iv) a poly-dT primer sequence were released and mixed with cell lysate and Master Mix. Barcoded, full-length cDNAs were then reverse-transcribed from poly-adenylated mRNA. Silane magnetic beads were used to remove leftover biochemical reagents and primers from the post GEM reaction mixture. Full-length, barcoded cDNAs were then amplified by PCR to generate sufficient mass for library construction. R1 (read 1 primer sequence) were added to the molecules during GEM incubation. P5, P7, a sample index, and R2 (read 2 primer sequence) were added during library construction via End Repair, A-tailing, Adaptor Ligation, and PCR. The final libraries contained the P5 and P7 primers used in Illumina bridge amplification. The Single Cell 3’ Protocol produced Illumina-ready sequencing libraries. A Single Cell 3’ Library comprised standard Illumina paired-end constructs which begin and end with P5 and P7. The Single Cell 3′ 16 bp 10 × Barcode and 10 bp UMI were encoded in Read 1, while Read 2 was used to sequence the cDNA fragment. Sample index sequences were incorporated as the i7 index read. Read 1 and Read 2 were standard Illumina^®^ sequencing primer sites used in paired-end sequencing.

### Quality control of scRNA-seq data

10X Genomics Cell Ranger software (version 3.1.0) was used to convert raw BCL files to FASTQ files, alignment and counts quantification. Briefly, reads with low-quality barcodes and UMIs were filtered out and then mapped to the reference genome. Reads uniquely mapped to the transcriptome and intersecting an exon at least 50% were considered for UMI counting. Before quantification, the UMI sequences would be corrected for sequencing errors, and valid barcodes were identified based on the EmptyDrops method [18]. The cell by gene matrices were produced via UMI counting and cell barcodes calling. The cell by gene matrices for each sample were individually imported to Seurat [19] version 3. 1. 1 for downstream analysis. Cells with unusually high number of UMIs (≥ 8000) or mitochondrial gene percent (≥ 10%) were filtered out. We also excluded cells with less than 500 or more than 4000 genes detected. Additionally, doublet GEMs also should be filtered out. It was achieved by using the tool DoubletFinder (v2.0.3) by the generation of artificial doublets, using the PC distance to find each cell’s proportion of artificial k nearest neighbors (pANN) and ranking them according to the expected number of doublets [20].

### General scRNA-seq data analysis

We employed a global-scaling normalization method “LogNormalize” that normalizes the gene expression measurements for each cell by the total expression, multiplies this by a scale factor (10,000 by default), and log-transforms the results. The formula is shown as follows: A gene expression level = log (1 + UMIA UMITotal × 10,000). To minimize the effects of batch effect and behavioral conditions on clustering, we used Harmony, an algorithm that projects cells into a shared embedding in which cells group by cell type rather than dataset-specific conditions, to aggregate all samples [21].

Integrated expression matrix is then scaled and performed on principal component analysis for dimensional reduction [22]. Then we implemented a resampling test inspired by the jackStraw procedure. We randomly permuted a subset of the data (1% by default) and rerun PCA, constructing a ‘null distribution’ of gene scores, and repeated this procedure. We identified ‘significant’ PCs as those who cng enrichment of low p-value genes for downstream clustering and dimensional reduction.

Seurat implements a graph-based clustering approach. Distances between the cells were calculated based on previously identified PCs. Briefly, Seurat embed cells in a shared-nearest neighbor (SNN) graph, with edges drawn between cells via similar gene expression patterns. To partition this graph into highly interconnected quasi-cliques or communities, we first constructed the SNN graph based on the euclidean distance in PCA space and refined the edge weights between any two cells based on the shared overlap in their local neighborhoods (Jaccard distance). We then cluster cells using the Louvain [23] method to maximize modularity. For visualization of clusters, the Uniform Manifold Approximation and Projection (UMAP) were generated using the same PCs.

### Transcription factor analysis

To carry out transcription factor network inference, analysis was performed on the SCENIC R package [24]. In brief, log-normalized expression matrix generated using Seurat was used as input, and the pipeline was implanted in three steps. First, we identified gene co-expression network via GENIE3 [25]. Second, we pruned each module based on a regulatory motif near a transcription start site via RcisTarget. Precisely, networks were retained if the TF-binding motif was enriched among its targets, while target genes without direct TF-binding motifs were removed. The retained networks were called regulons. Third, we scored the activity of each regulon for each single cell via the AUC scores using AUCell R package.

### Pseudotime trajectory analysis

To investigate cellular differentiation trajectories, the Slingshot algorithm was employed, a computational method specifically developed for inferring pseudotemporal ordering and lineage relationships from single-cell RNA sequencing data. The underlying cellular structure was first identified through dimensionality reduction, followed by clustering of cells based on their transcriptional profiles. Subsequently, the differentiation process was modeled as a continuous trajectory, with branch points being computationally determined to represent potential lineage bifurcations.

### Cellular interaction analysis

Single-cell datasets provide a unique opportunity to analyze intercellular communication mediated by ligand‒receptor interactions. CellChat includes a repository of ligand‒receptor interactions and a statistical framework for predicting enriched interactions between two cell types from single-cell transcriptomic data. The interactions between cell types are calculated based on ligand-receptor co-expression using the CellChat tool, with default settings and log-transformed SCTransform normalized counts as input. The interactions between immune regulatory MSCs from patient samples and other functional subpopulations were analyzed and compared with interactions in healthy individuals. To calculate the average number of interactions, we summed all significant (p-value < 0.05) interactions between the two cell types for each patient/donor and averaged this number across patients/donors. To calculate the interaction strength between the two cell types among patients, we summed the average expression of all ligand-receptor pairs calculated by Cellchat. For the interaction analysis of the HMGh-MSC subset, all cells of specific cell types from all patients/donors were aggregated, and networks were generated separately for healthy donors and NDMM patients. In this network, cell types are nodes, with their top four interaction partners displayed as connecting edges, weighted by average strength and the number of edges.

### Construction of prognostic model and survival analysis

The expression profile and survival information of patients from the GSE4581, GSE136337 and the TT3 treatment cohort from the GSE24080 datasets were also downloaded from Gene Expression Omnibus (GEO; https://www.ncbi.nlm.nih.gov/geo/) database. Data on DEGs obtained from HMGh cells versus other cell types, with GSE136337 as the training set and GSE4581 as the validation set. The TT3 treatment cohort from the GSE24080 dataset was used to assess the applicability of the prognostic model in relapsed and refractory multiple myeloma. Prognostic models were constructed using univariate Cox regression analysis (p < 0.05) and the least absolute shrinkage and selection operator (LASSO) regression analysis by the “glmnet” and “survival” R packages. Risk scores for each sample were calculated based on the LASSO model, and the model's predictive performance was evaluated using Harrell’s C-index by the “Hmisc” R package. Pairwise C-index comparisons were conducted using compareC, with significance denoted as p < 0.05 (*), p < 0.01 (**), and p < 0.001 (***). MM tumor samples were divided into high-risk and low-risk groups based on the cutpoint value, determined by the surv_cutpoint function in the “survminer” R package. Survival analysis was performed using Kaplan–Meier curves and log-rank tests (p < 0.05) by the “survival” R package. The receiver operating characteristic (ROC) curve was used to predict the score by the disturbance scoring model using the “timeROC” R package. Multivariate Cox regression analysis was used to evaluate the combined impact of risk scores and other clinical features (e.g., gender, age, ISS stage) on survival time by the “survival” R package. Forest plots were generated with the “forestplot” function in the “forestplot” R package to display the hazard ratios (HR) and 95% confidence intervals (CI) of each clinical variable in the multivariate Cox regression analysis.

The Wilcoxon signed-rank test was used to compare the differences between the two groups of samples, and the Kruskal–Wallis test was used to compare the differences between more than two groups of samples. NS indicates p > 0.05, * indicates p < 0.05, ** indicates p0.01, *** indicates p < 0.001, and ***indicates p < 0.0001.

## Results

### Mesenchymal stem cells (MSCs) exhibit notable heterogeneity in the progression of multiple myeloma

**Fig. 1:**
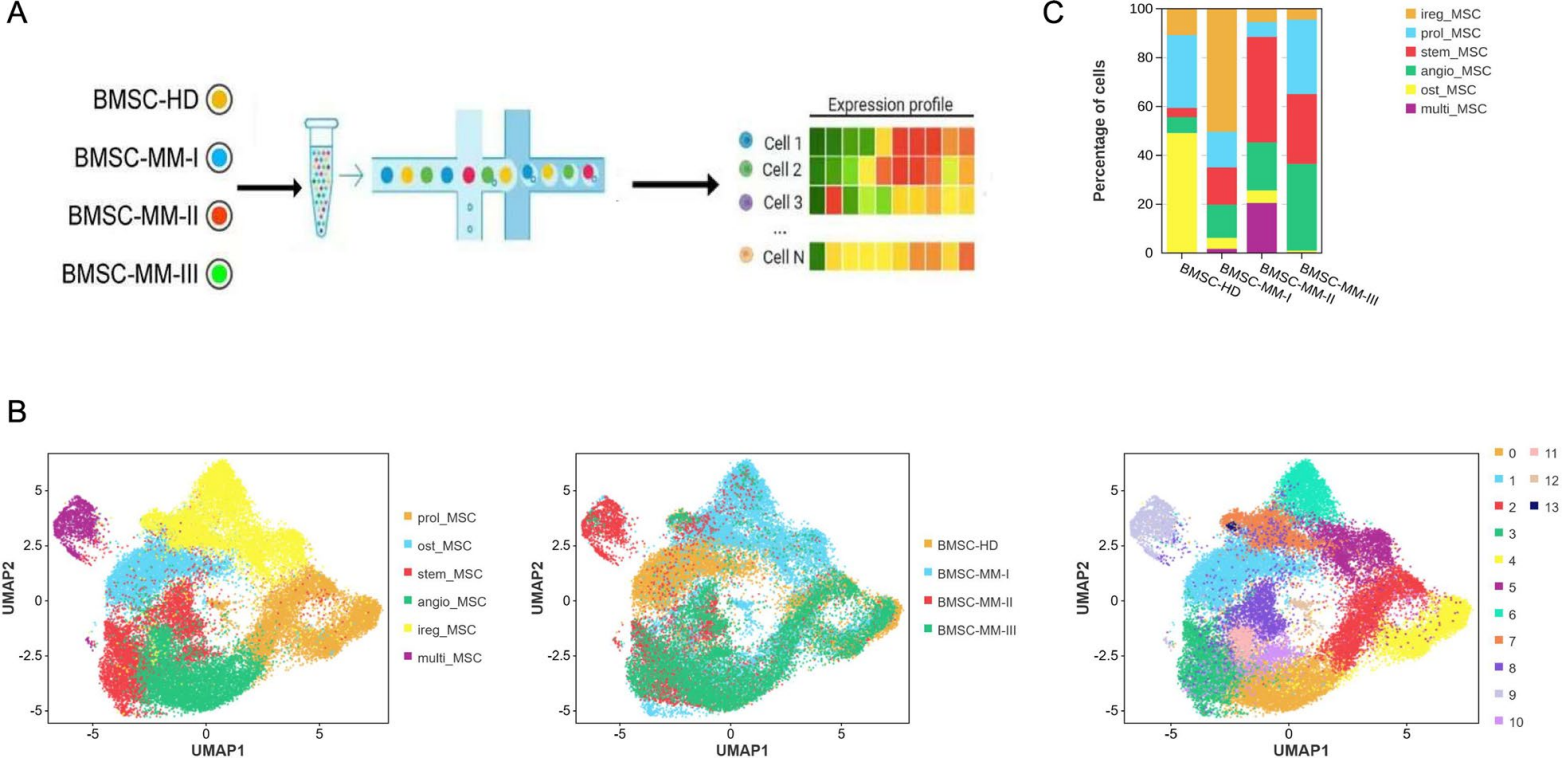
Single-cell RNA-seq reveals heterogeneity of Mesenchymal Stem Cells in normal bone marrow and multiple myeloma BM tissues. **A** Illustration of workflow of scRNA-seq in human normal bone marrow and multiple myeloma samples. **B** UMAP visualization of color-coded clustering of 31,766 cells reveals 6 cellular clusters. The general identity of each cell cluster is shown on the right. **C** The proportion of cell lineages in multiple myeloma BMSC and normal BMSC

To elucidate the distinct characteristics of MSCs within this disease context, patient-derived bone marrow from various R-ISS stages of multiple myeloma was collected and sorted with magnetic beads. Single-cell sequencing was subsequently performed utilizing the 10 × Genomics Chromium platform (Fig. 1A). After stringent quality control measures to filter out low-quality cells and remove apparent doublets, we obtained the transcriptomes of 31,766 viable cells (Fig. 1B, S1 A).

Next, we conducted unsupervised clustering on the isolated single cells while addressing batch effects through the Harmony algorithm, ultimately identifying 14 distinct cell clusters labeled from 0 to 13. By defining characteristic marker genes and performing functional enrichment analysis on highly differentially expressed genes (F.S1B), it was discovered that the C3-8–10 subgroup highly expresses GAS1 and DPP4, whereas the C2-4–12 subgroup highly expresses TOP2 A and MKI67. The DEGs of the other subgroups were enriched in different pathways, on which we based our clustering. We finally delineated six primary cell clusters, namely, the stemness cluster (C3-8–10), proliferation cluster (C2-4–12), and four specific functional clusters, categorized as osteogenic (C1), angiogenic (C0-11), immune regulatory (C5-6–7-13), and multipotent (C9) clusters, as depicted by UMAP plots (Fig. 1B). Furthermore, the proportions of these clusters varied across different disease stages (Fig. 1C; S1 C). Notably, the immunoregulatory MSC cluster exhibited substantial variation in R-ISSI samples, with marked aggregation. Healthy BM samples displayed a predominance of osteogenic subclusters; conversely, angiogenic subclusters were significantly enriched in samples progressing toward the end stage. These characteristics are consistent with the clinical manifestations of the progression of multiple myeloma. Moreover, we identified sets of differential gene expression profiles among these various functional subclusters (F.S1D-E).

Thus, our findings showed that bone marrow mesenchymal stem cells demonstrate pronounced heterogeneity across different stages of multiple myeloma on the basis of their expression levels at single-cell resolution.

### Immune regulatory MSCs exhibit immunosuppressive functions in the early stages

MSCs mainly regulate immunity through the release of soluble cytokines and/or cell-to-cell contact. Various cytokines related to immune regulation include interleukins, prostaglandins, and indoleamine 2,3-dioxygenase, among others. Considering the characteristics of the quantity of MSCs in R-ISSI samples, we speculate that the prephase acute inflammatory response mediated by immunoregulatory MSCs is significant.

**Fig. 2:**
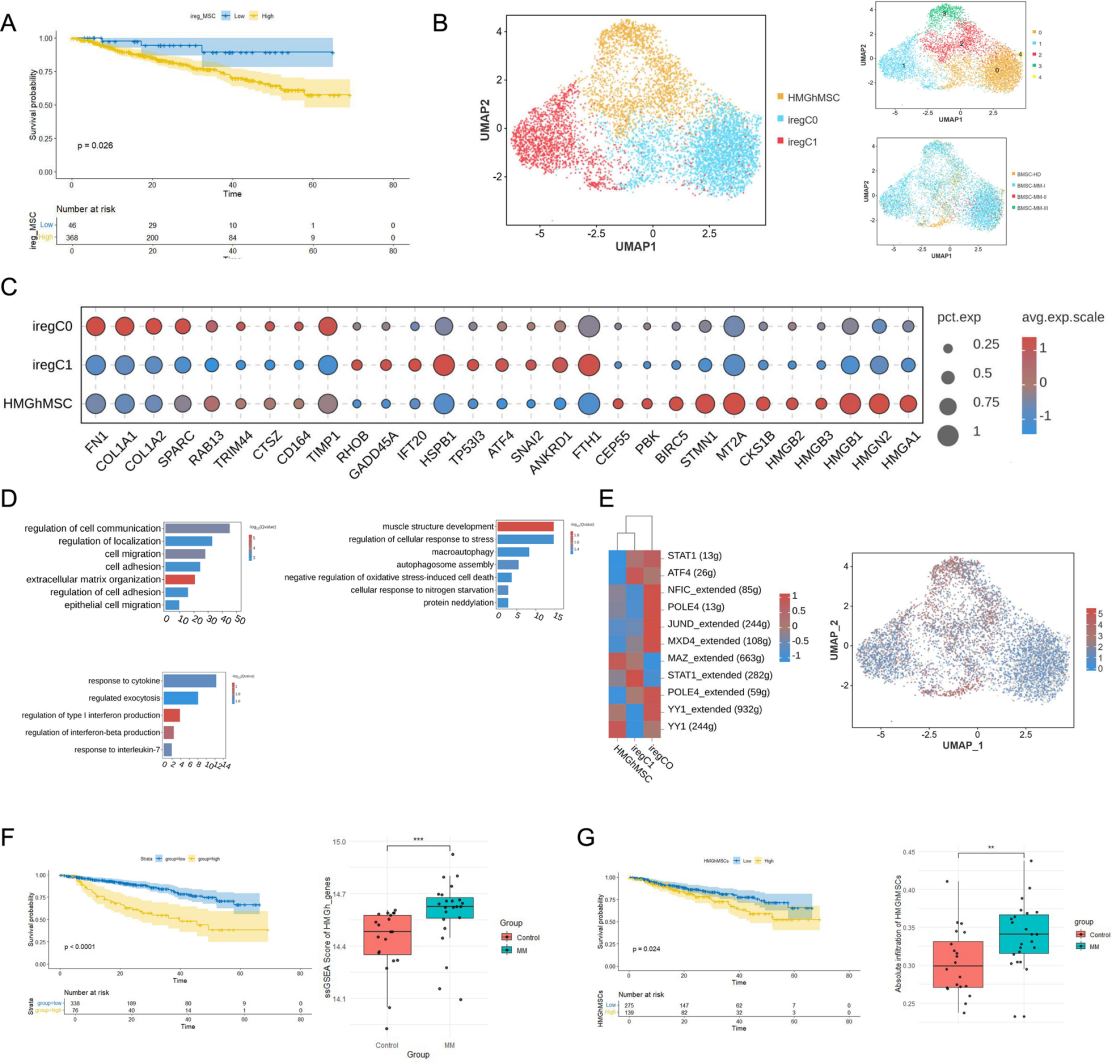
Single-cell landscape of iregMSCs in multiple myeloma at different stages. **A** Kaplan–Meier survival analysis of iregMSCs in multiple myeloma (MM) based on the GSE4581 dataset. **B** UMAP plot showing iregMSC subpopulations. **C** Dot plot showing the expression of representative genes in iregMSC subpopulations. **D** The Gene Ontology (GO) analysis of iregMSC subpopulations: iregCO, iregC1, and HMGhMSC (left to right). **E** Transcription factor (TF) analysis of iregMSC subpopulations and FeaturePlot of HMGN2 expression. **F** Kaplan–Meier survival analysis of the HMGh genes (GSE4581) and its high expression in MM (GSE24870). **G** Kaplan–Meier survival analysis of the HMGhMSC subpopulation (GSE4581) and its high infiltration proportion in MM (GSE24870)

We correlated our single-cell sequencing results with clinical information and used the deconvolution algorithm CIBERSORTx [26] to simulate cell type-specific gene expression profiles and predict the abundance of MSCs defined by scRNA-seq in the external chip dataset from the GSE4581 cohort. The robustness of CIBERSORTx in predicting cell type-specific gene expression profiles from GEO datasets was tested by training on our own scRNA-seq dataset (FS2 A-B). Next, to assess the prognostic value of MSCs in multiple myeloma, we examined their correlation with the overall survival (OS) of MM patients. The results revealed that increased MSC abundance was associated with poor OS in the GSE4581 cohort (p = 0.026) (Fig. 2A). This finding is consistent with the reported characteristics of MSCs in TME regulation, which largely suppress the activation of immune cells. Therefore, we propose that MSCs may significantly participate in remodeling the immunosuppressive microenvironment in myeloma.

To better understand the potential role of MSCs in immune regulation, we performed unsupervised clustering of these cells, classifying them into three subclusters (Fig. 2B). We identified the DEGs of each subunit and presented the top 5 genes with the most significant differences using a heatmap (FS2 C). Each subcluster has different characteristics: compared with the other subclusters, the C0 cluster expresses high levels of FN1, COL1 A1, COL1 A2, and SPARC, all of which are related to the extracellular matrix (Fig. 2C). MSCs may remodel the extracellular matrix, thereby creating a barrier effect on the infiltration of drugs and immune cells. In tumor-associated inflammation, chemokines are key participants in the recruitment of BM-MSCs to the site of inflammation. We speculate that in the bone marrow microenvironment, MSCs are recruited to the tumor periphery, where they promote solidification of the tumor extracellular matrix by secreting large amounts of collagen and fibronectin and inhibiting the infiltration of immune cells and the penetration of antitumor drugs recruited by tumor-secreted chemokines. The peripheral levels of TRIM44, CTSZ, CD164, and TIMP1 can stimulate granulocyte production in mouse bone marrow, and high levels of TIMP1 are positively correlated with increased immune infiltration levels of tumor-infiltrating lymphocytes. Additionally, COL1 A1, COL1 A2, and MYLK are markers of fibroblasts (myofibroblasts), which indicates that MSCs may also be reprogrammed by tumor cells into cancer-associated fibroblasts (CAFs), further assisting in malignant progression. Furthermore, MSCs can induce epithelial‒mesenchymal transition. These cellular transformations are closely related to tumor-induced inflammation and function as important exogenous factors in malignant cell transformation within the cancerous tissue niche.

C1 cluster functional enrichment revealed significant enrichment in pathways related to autophagosome assembly, macroautophagy, negative regulation of oxidative stress-induced cell death, and protein neddylation. The DEGs included RHOB, GADD45 A, IFT20, TP53I3, SNAI2 and ANKRD1. The involvement of SNAI2 in epithelial‒mesenchymal transition (EMT) serves as a further example of the complex regulatory networks at play during muscle development and stress responses. TP53I3 and RHOB are involved in cell senescence and programmed cell death. GADD45 A, another gene of interest, has been implicated in the regulation of the cell cycle and apoptosis in response to stress. Its role in promoting cell cycle arrest during oxidative stress highlights its potential as a therapeutic target.

Clusters C2 and C3 highly upregulated the expression of high mobility group proteins, including HMGB2, HMGB3, HMGB1, HMGN2, and HMGA1 (Fig. 2C). High-mobility group proteins are the most abundant group of chromatin proteins in eukaryotic cells after histones. HMGB1, a classic inflammatory damage molecule, is released by dying tumor cells, and DAMPs, which bind to TLR4 or RAGE innate immune receptors on DC cells, mediate innate immune recognition of cancer. Here, we defined the unique MSC group as also secreting HMGB1 and high-mobility group proteins, suggesting that they may similarly influence the immune recognition of tumors mediated by innate immunity. Based on the above findings, we named these cells HMGhMSCs. Additionally, these cells exhibit specific upregulation of CEP55, PBK, BIRC5, STMN1, MT2 A, and CKS1B, all of which are related to tumor inflammatory responses. We subsequently conducted functional enrichment, and the results revealed that MSCs were significantly enriched in cytokine-mediated immune responses, including the cellular response to IL-1- and IL-12-mediated signaling pathways, while also revealing macromolecular complex assembly, intracellular protein transport, exocytosis, vesicle-mediated transport, and Golgi vesicle transport (Fig. 2D). These data suggest that HMGhMSCs play a significant role in the early stages of multiple myeloma through paracrine signaling. Inflammatory cytokines such as TNF-α and IL-1β enable MSCs to release high levels of CCL2, CXCL8, and CCL5, leading to exacerbated inflammation and procancer characteristics. Tumor-induced MSCs can further activate and release chemoprotective and immunomodulatory factors, including CXCL1, CXCL2, and IL-8, which are beneficial for tumor progression, among which MSCs produce CXCL2, VEGF, TGF-β, and IL-6, which can further increase tumor invasiveness by promoting tumor angiogenesis.

Our Slingshot pseudotime analysis revealed that HMGhMSCs (C2/C3) occupy an intermediate position along the differentiation trajectory (FS2 F-G). Given the significant functional, transcriptional, and tumor microenvironment-regulatory differences between HMGhMSCs and iregC0/C1, we hypothesize that HMGhMSCs may serve as a critical dynamic intermediate state, bridging ECM remodeling and inflammatory signaling. The ECM-reprogramming activity of C0 may induce MSC transition toward HMGhMSCs via mechanical or biochemical signals. Subsequently, HMGhMSCs amplify inflammatory responses through HMGB1 and cytokine secretion (e.g., IL-6, CXCL8), thereby promoting C1’s stress-adaptive programs, such as oxidative stress-induced autophagy. We further propose that HMGhMSCs may originate from a rare pre-existing MSC subset intrinsically expressing high levels of HMGB1/2/3 and pro-inflammatory genes. Under homeostatic conditions, these cells could function as sentinels for tissue damage. However, in the inflammatory tumor niche, chemokines and tumor-derived factors may selectively expand this population. Thus, HMGhMSCs act as a pivotal transitional node, driving the shift from C0 (representing the initial ECM-remodeling phase) toward C1 (reflecting stress adaptation), while amplifying inflammatory signals to shape the immunosuppressive tumor microenvironment.

Next, we explored the transcription factors (TFs) that may be involved in immune regulation in mesenchymal stem cells. We used SCENIC19 and predicted several potential regulatory genes that are upregulated in MSCs. We found that some of these TFs, such as YY1, STAT1, MAZ, JUND, POLE4, MXD4, NFIC, and ATF4, may have the ability to regulate the observed cytokine secretion genes and immune regulatory genes (Fig. 2E). In addition, STAT1, a classical transcription factor that participates in the JAK/STAT pathway or cascades with the RIG1, NF-kappaB, and IRF signaling pathways, has been reported to mediate tumor immunity in multiple myeloma. All these transcription factors are highly expressed in HMGhMSCs, suggesting that immune suppression is potentially regulated by the unique MSCs we identified.

We previously explored the relationship between complete immunoregulation by MSCs and patient prognosis and reported that increased infiltration of these cells is associated with poor prognosis. For further investigation, we utilized CIBERSORTx to predict the abundance of subclusters we defined in the GSE4581 and GSE24870 cohorts. The results indicated that a higher HMG protein content was associated with poor prognosis (Fig. 2F), similar to the significant infiltration level of HMGhMSCs in the tumor group compared with that in the control group (Fig. 2I). These results emphasize the predictive potential of HMGhMSCs in the prestage of multiple myeloma.

### Membrane proteins are significant in immune regulatory HMGhMSCs

Based on the discovery of a unique subset of HMGh-MSCs, we further investigated specific genes involved in MM progression. Therefore, we compared the intergroup differences between different samples. Group comparisons between MM patients and healthy individuals revealed various transcriptional differences.

**Fig. 3:**
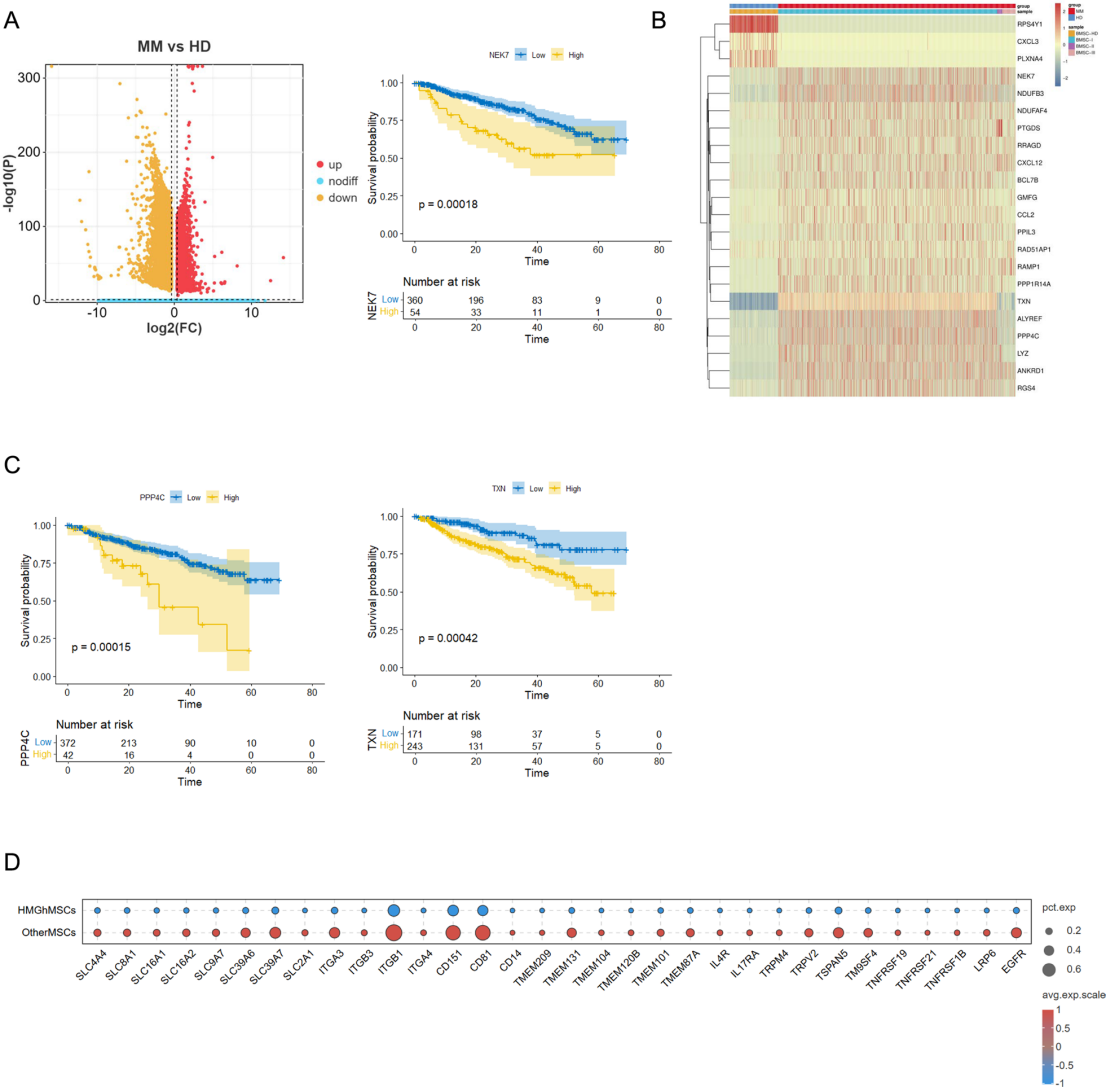
HMGhMSC subpopulation in multiple myeloma at different stages. **A** DEGs of HMGhMSC subpopulations in MM and HD. **B** Heatmap showing the expression of representative prognostic DEGs in iregMSC subpopulations during MM progression and HD. **C** Kaplan–Meier curve of overall survival of TXN, PPP4 C, and NEK7 in MMbased on the GSE4581. **D** Dot plot showing the downregulation of membrane proteins involved in Wnt signaling and cell communication in MM-HMGhMSCs compared to HD

We identified thousands of differentially expressed genes (DEGs) in MM patient samples, which can be divided into different clusters and functional pathways (Fig. 3A), including inflammation-related signaling pathways (wnt/mTOR/MAPK), immune cell regulation (macrophage/T cells/NK cells), EMT, protein folding, and mRNA splicing. Combined with GEO chip data and filtering by |logFC|> 2, we identified a total of 132 upregulated genes in MM-HMGhMSCs, among which 45 were associated with poor prognosis and 23 with improved prognosis (Fig. 3B), including LYZ, RRAGD, PPP1R14 A, RAMP1, ANKRD1, PTGDS, GMFG, PPIL3, ALYREF, CXCL12, and CCL2. Specifically, GMFG, TXN, PPP4 C, and NEK7 have been reported to be related to inflammatory responses [27, 28], whereas the significant upregulation of NDUFB3 and NDUFAF4 is associated with mitochondrial ROS production. In addition, PTGDS is involved in the wnt signaling pathway, BCL7B affects chromatin accessibility, PPIL3 accelerates protein folding, RGS4 regulates G protein-coupled receptors, ALYREF mediates RNA m5c modification, ASF1B functions as a histone chaperone that resists gene silencing, and RAD51 AP1 is related to DNA damage repair. These significantly upregulated genes were strongly correlated with poor prognosis (Fig. 3C), further confirming that HMGhMSCs are responsible for immune suppression in myeloma.

However, more genes were downregulated in patients with HMGhMSCs (total of 859), of which 263 genes were associated with improved prognosis and 188 with poor prognosis (Fig. 3B). Consistent with the function of HMGhMSCs, many of these genes, such as RPS4Y1, CXCL3 and PLXNA4 [29, 30], are related to triggering inflammatory responses. Interestingly, many conspicuous membrane proteins, including the SLC family members SLC4 A4, SLC8 A1, SLC16 A1/2/7, SLC9 A7, SLC39 A6/7, SLC2 A1, the integrin family proteins ITGA3/B3/B1/A4, the CD family proteins CD13/151/81, the TMEM family proteins TMEM209/131/104/120B/101/87 A, the interleukin receptors IL4R/IL17RA, the membrane ion channels TRPM4/TRPV2/KCNMA1, the four transmembrane proteins TSPAN5/TM9SF4, the tumor necrosis factor superfamily proteins TNFRSF19/TNFRSF21/TNFRSF1B, and LRP6, which are involved in the Wnt signaling pathway, as well as the epidermal growth factor receptor EGFR (Fig. 3D), were also downregulated. Compared with those in HD samples, these membrane proteins are significantly downregulated in MM-HMGhMSCs and are closely related to prognosis. Given the crucial role of membrane proteins in cell-to-cell communication, we speculate that HMGhMSCs communicate with other cells in their microenvironment through ligand molecules and membrane protein receptors. This interaction may reduce the recruitment of immune effector cells and suppress their activity, ultimately creating an immunosuppressive microenvironment in the bone marrow.

The abundance of differentially expressed genes in MM-HMGhMSCs supports our hypothesis that this unique subset can regulate cytokine–receptor signaling and mediate immunosuppressive processes. Moreover, genes encoding inflammation-related proteins and membrane proteins should be emphasized, as they may be potential targets for reversing the immune-suppressive microenvironment mediated by HMGhMSCs in myeloma.

### Ligand‒receptor interaction networks between HMGhMSCs and other subsets in the bone marrow potentially affect the tumor microenvironment

**Fig. 4:**
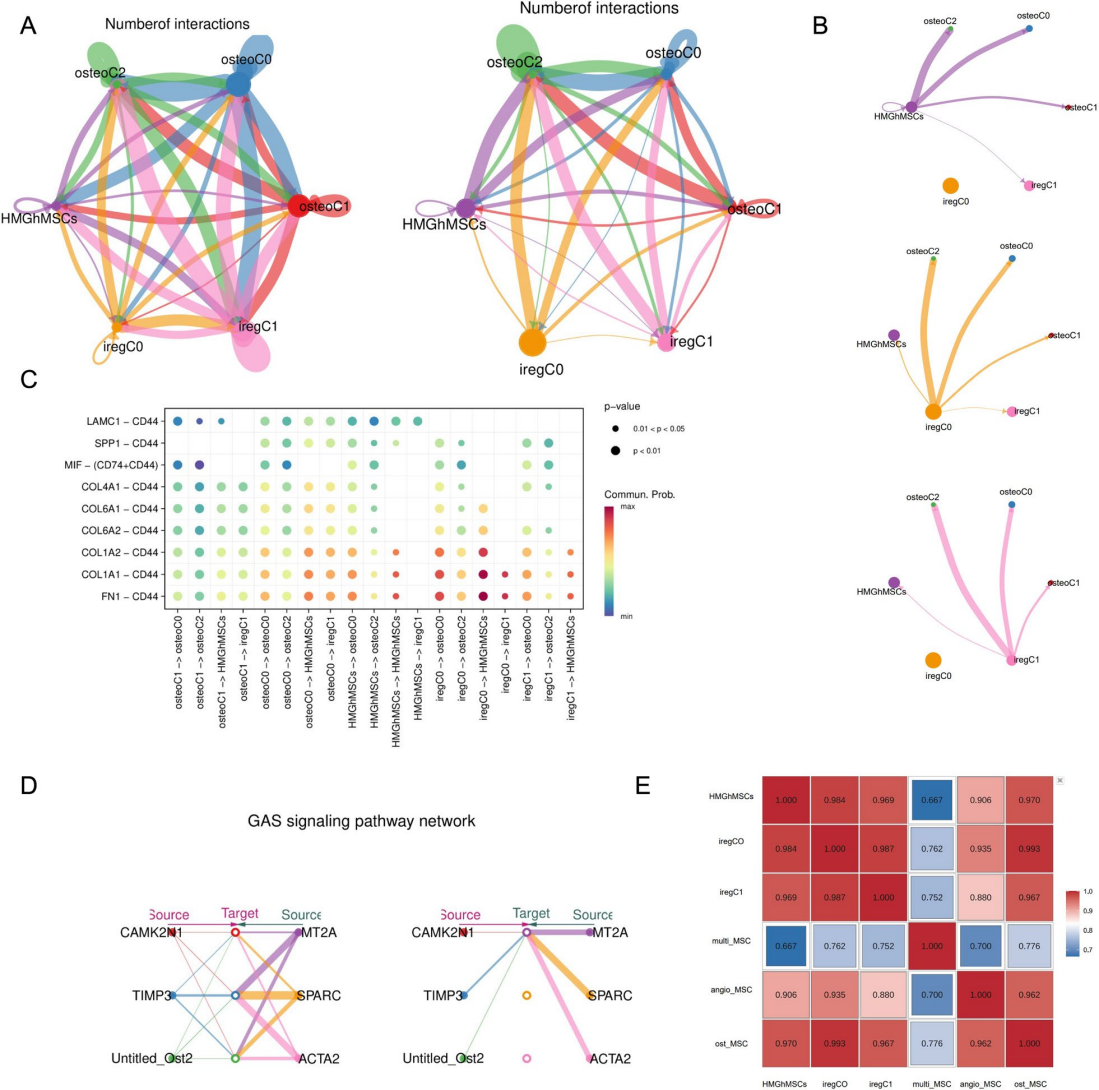
Cell communication network between HMGhMSCs and other subpopulations in the bone marrow. **A** Cell–cell communication between iregMSC and ostMSC in HD (left) and MM patients (right). **B** Cell–cell communication between iregMSC and ostMSC in MM patients. **C** Cell–cell communication between single-subpopulation iregMSC and ostMSC in MM patients. **D** The GAS signaling pathway network in MM-I patients. Left and right portions show the autocrine and paracrine signaling, respectively. **E** Heatmap showing the correlation between HMGhMSC and other subpopulations

Generally, we observed a dense communication network between HMGhMSCs and other cells, and HMGhMSCs were more commonly found as ligands that regulate other cells. We found that the interaction between HMGhMSCs and osteogenic MSCs in MM patient samples was significantly greater than that in HD samples (Fig. 4A), which was consistent with the high accumulation of HMGhMSCs in the early stages of myeloma. In addition to HMGhMSCs, other immunoregulatory MSC subgroups also regulate osteogenic MSCs (Fig. 4B).

Next, we identified ligand‒receptor pairs between HMGhMSCs and other subclusters. We found that the communication of HMGhMSCs with other cells occurs mainly through the ECM-receptor, secreted signaling, and infrequent cell–cell contact. Regardless of whether HMGhMSCs act as a source subset or target, CD44 appears to be the major ligand‒receptor molecule (Fig. 4C). In the context of the crosstalk between tumor cells, stromal cells, and immune cells, the interplay between CD44 and SPP1 has been proposed to suppress CD8 + T-cell activation and foster tumor immune tolerance and evasion [31]. CD44 also facilitates lymphocyte infiltration, macrophage polarization, and the differentiation of mesenchymal stem cells (MSCs) into cancer-associated fibroblasts (CAFs) [32–35]. Furthermore, the CD44‒SPP1 axis is vital for cell‒cell communication and exerts significant immunomodulatory effects within the tumor microenvironment (TME). In particular, L-R pairs formed by the combination of CD44 and collagen family genes (COL1 A1, COL1 A2, COL4 A1, COL4 A2, COL6 A1, COL6 A2 and COL6 A4) and the adhesion protein-encoding gene family (LAMB1, LAMB2, and LAMC1) were highly active in HMGhMSCs and osteogenic MSCs.

In addition, GAS6-AXL has attracted our attention, as immune-regulatory MSCs exhibit a strong GAS6 regulatory effect (Fig. 4D). The Gas6/AXL pathway regulates angiogenesis, immune-related molecular markers, and the secretion of certain cytokines (MHC-I, PD-L1, IL-4, CCL3-5, and G-CSF) in the tumor microenvironment, and it also regulates the functions of various immune cells (NK, DC, M2, and Treg) [36]. AXL expression has been detected on bone marrow-derived cells (BMDCs), dendritic cells (DCs), macrophages, monocytes, and natural killer (NK) cells. Increasing evidence suggests that the interaction between abnormal physiological factors in tumors, host immune cells, and the TME may upregulate AXL and Gas6 in the presence of myeloid-derived suppressor cells (MDSCs) or hypoxic environments, thereby promoting a protumor microenvironment. Therefore, AXL may be a key mediator in the malignant microenvironment of tumors.

The results of the Spearman correlation analysis of these cells also confirmed that HMGhMSCs are strongly correlated with other immunoregulatory MSCs and osteogenic MSCs (Fig. 4E). These interactions between cells reveal the complex dynamic changes in the molecular characteristics of HMGhMSCs, which may affect bone damage and angiogenesis in MM. The molecular characteristics and functional crosstalk of MSC subunits are closely related.

### Prognostic model for MM patients on the basis of HMGhMSC subgroup characteristics

**Fig. 5:**
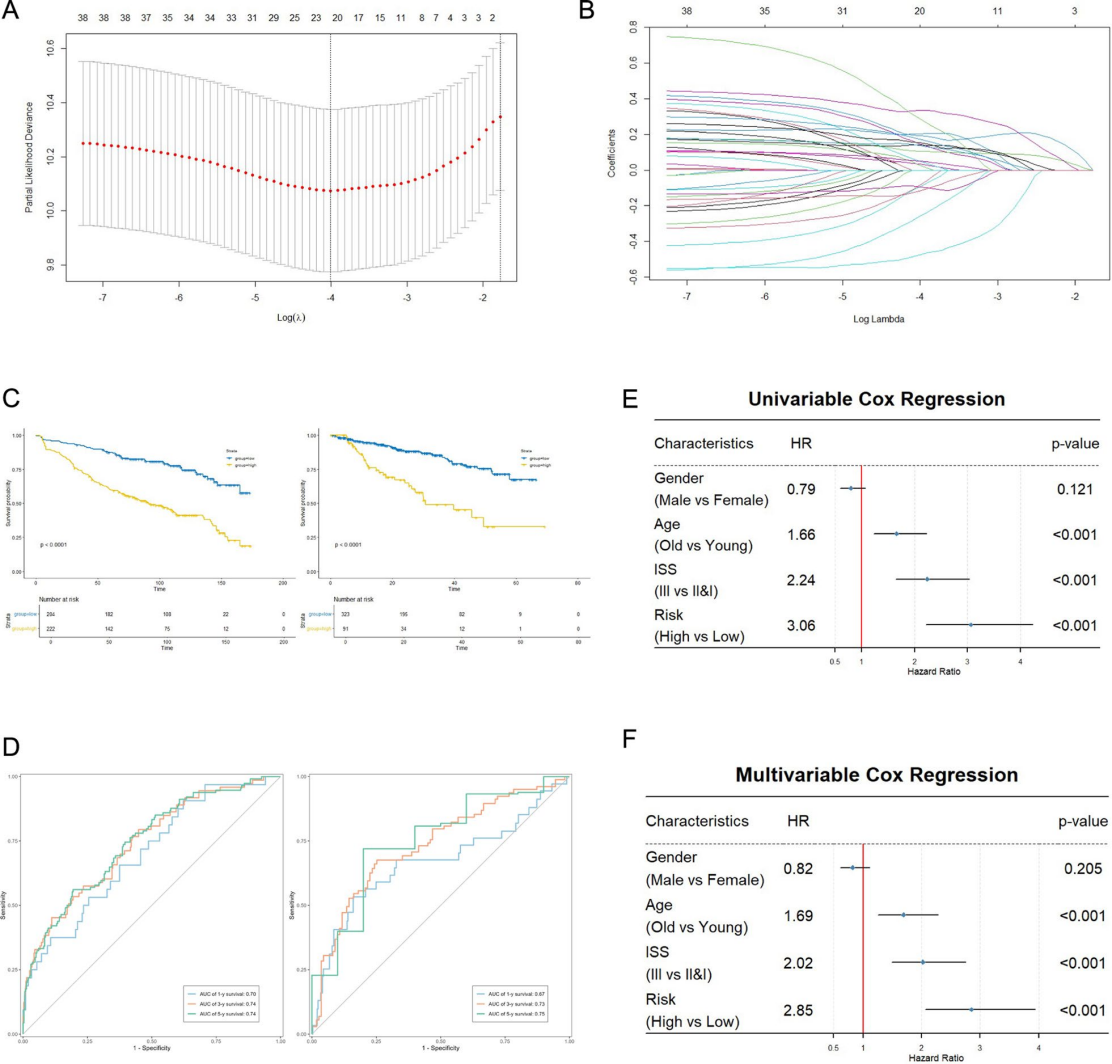
Establishment and Evaluation of a Prognostic Model Based on the HMGhMSC subpopulations. **A** Confidence interval under each lambda in LASSO regression. **B** Change in trajectory of the LASSO regression independent variable. **C**, **D** The performance of th.e model in different cohorts. The survival curve of patients in high- and low-risk groups (c) and 1-, 3-, and 5-year time-dependent ROC curves of models from the GSE136337, and the GSE4581 cohorts, respectively (d)

Based on the above findings, we attempted to evaluate the independent predictive value of HMGhMSC gene characteristics. A total of 220 genes from this cell subgroup, including genes that are highly expressed in HMGhMSCs compared with other cells (FDR < 0.05, fold change > 2, min.pct > 0.25) and genes that are highly expressed in HMGhMSCs, were used to construct the prognostic model. The GEO database, specifically the GSE136337 cohort, was selected as the training set. Among these genes, 41 genes were identified as prognostically relevant through univariate Cox regression analysis (p < 0.05). LASSO regression analysis was performed on the training set to remove redundant genes, with the number of seeds set to 10,210. A total of 20 genes associated with the prognosis of multiple myeloma patients were identified, and the results are shown in Fig. 5A, B and Fig. S3 A. We plotted Kaplan‒Meier survival curves for these 20 genes and found significant differences in the KM curves, as shown in Fig. S3B. The model’s predictive performance was evaluated using Harrell’s C-index with the “Hmisc” R package, which yielded a C-index of 0.70, indicating the strong predictive ability of the model.

Based on the prognostic model's score, the MM tumor samples were stratified into high-risk and low-risk groups. To validate the model’s stability, we plotted survival curves in the GSE136337 and GSE4581 cohorts, with GSE4581 serving as the validation set. The results revealed that the prognosis of patients in the high-risk group was significantly worse than that of patients in the low-risk group, a trend observed in both cohorts, as shown in Fig. 5C. Gene expression characteristics and survival status of multiple myeloma patients in different groups, as shown in Fig. S3 C, D. ROC curves were used to assess the model’s effectiveness in predicting patient prognosis. In the GSE136337 training set, the AUCs for 1-, 3-, and 5-year survival were 0.70, 0.74, and 0.74, respectively. In the GSE4581 cohort, the AUCs for 1-, 3-, and 5-year survival were 0.67, 0.73, and 0.75, respectively. To further evaluate the applicability of the prognostic model in patients with relapsed and refractory multiple myeloma, we analyzed the TT3 treatment cohort from the GSE24080 dataset. The model’s performance was assessed in terms of event-free survival (EFS) and overall survival (OS). The area under the curve (AUC) values for EFS at 1-, 2-, and 3-year intervals were 0.77, 0.76, and 0.79, respectively. For OS, the corresponding AUCs were 0.75, 0.73, and 0.75. Notably, patients classified as high-risk exhibited significantly poorer prognoses, as shown in FS3E-F. Overall, these findings indicate that our prognostic model performs well across different cohorts (Fig. 5D).

To verify whether the RS can serve as an independent prognostic factor, univariate and multivariate Cox regression analyses were conducted on the clinical characteristics of patients (such as age, sex, and ISS). The results showed that regardless of the type of Cox regression analysis used for statistical testing, the RS is an independent prognostic risk factor for patients, and this scoring model has a higher hazard ratio (HR) than other factors do (Fig. 5E, F). A comparative analysis was conducted between the proposed prognostic model and the International Staging System (ISS). The results demonstrated that the model achieved a significantly higher concordance index (C-index) compared to ISS, and exhibited superior area under the curve (AUC) values at 1-, 3-, and 5-year survival time points, indicating enhanced discriminatory capability, as shown in FS3G-H. When combined with the ISS, the model demonstrated improved predictive stability, suggesting a complementary relationship between molecular and clinical staging parameters. The model may partially capture key molecular features associated with disease progression and drug resistance in multiple myeloma, thereby contributing to its biological interpretability and clinical relevance.

### The tendency of MSC osteogenic differentiation to be related to osteolysis in MM

**Fig. 6:**
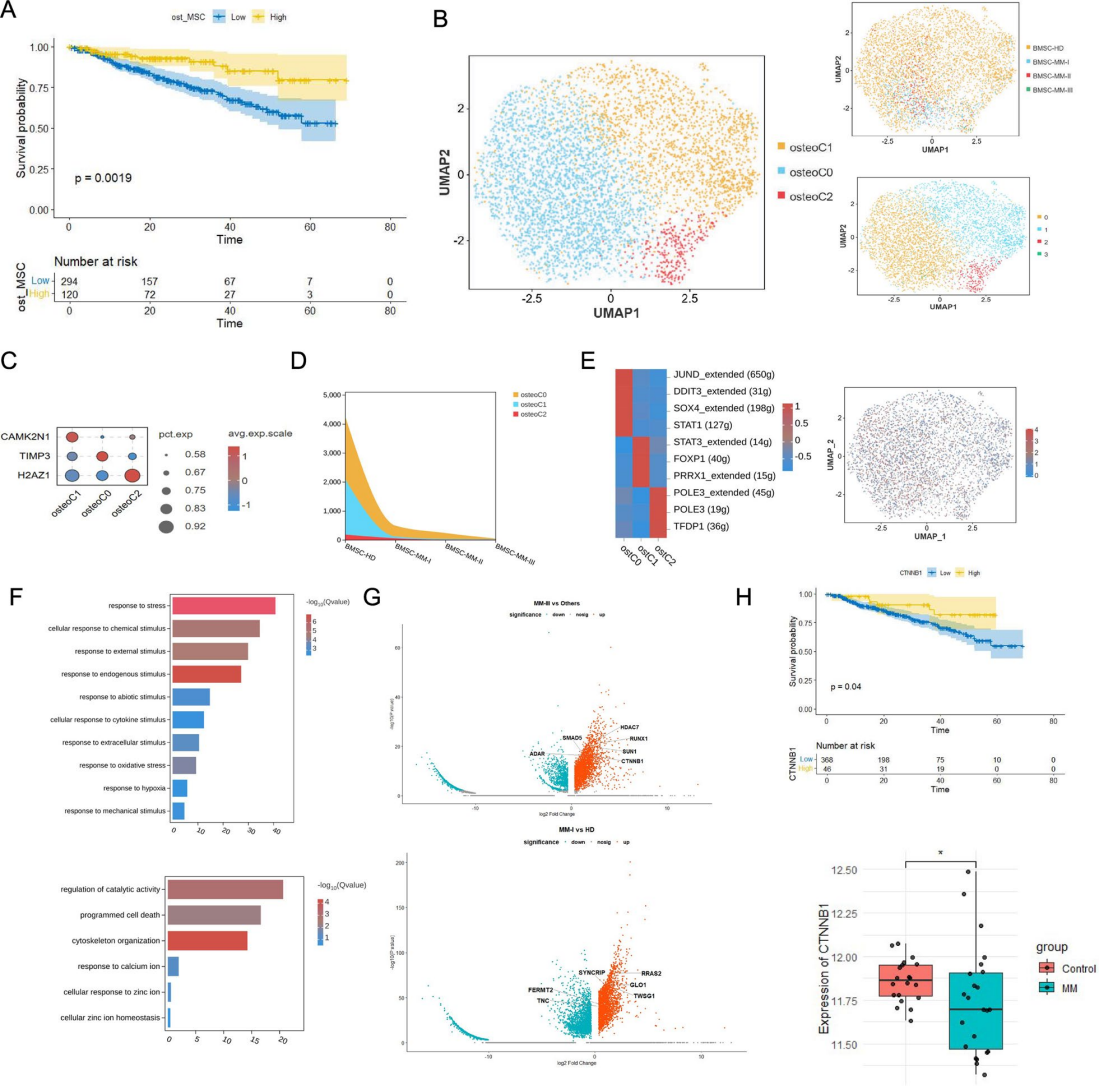
Single-cell landscape of osteogenic MSCs in multiple myeloma at different stages. **A** Kaplan–Meier survival analysis of osteogenic MSCs in multiple myeloma (MM) based on the GSE4581 dataset. **B** UMAP plot showing osteogenic MSC subpopulations. **C** Dot plot showing the expression of representative genes in osteogenic MSC subpopulations. **D** The numbers of osteogenic MSC subpopulations in multiple myeloma progression. **E** Transcription factor (TF) analysis of osteogenic MSC subpopulations and FeaturePlot of JUND expression. **F** The Gene Ontology (GO) analysis of osteogenic MSC subpopulations: ostC0 and ostC1. **G** DEGs of osteogenic MSC subpopulations in MM-III vs other patients and MM-I vs HD. **H** Kaplan–Meier survival analysis of the CTNNB1 (GSE4581) and its high expression in MM (GSE24870)

Patients with multiple myeloma always suffer severe osteolysis, and mesenchymal stem cells are known to differentiate into osteoblasts. Thus, we propose that MSCs may be related to bone damage in myeloma. Consistent with our hypothesis, the number of osteogenic MSCs was highest in HD samples and significantly decreased with disease progression (Fig. 6A). Therefore, we used the deconvolution algorithm CIBERSORTx to assess the abundance of osteogenic MSCs in the GSE4581 cohort and found that a greater proportion of osteogenic MSCs was associated with longer overall survival in multiple myeloma patients (p = 0.0019) (Fig. 6B). Similar to the other functional MSC clusters, we performed unsupervised classification of the osteogenic MSCs and defined three subunits, the C0, C1, and C2 subunits (Fig. 6C-D), among which the C0 cluster accounted for the largest proportion (FS4 A).

We found that C0 MSCs highly expressed GOF15, ACTA2, PSAT1, PTX3, and IGFBP7, and functional enrichment of these DEGs revealed a close relationship with internal and external stimuli, such as hypoxia, endoplasmic reticulum stress, and DNA damage (Fig. 6E), which suggests that this group of MSCs is stimulated by tumor cells to a large extent, affecting their tendency to differentiate into osteoblasts. On the other hand, C1 showed differential expression of WNT7B, CRYBG1, and PITPNC1 (FS4B-C), while ion binding and calcium channels were significantly enriched. Ca2 + is known to play a crucial role in the osteogenic tendency defined by intracellular calcium oscillations (the process by which Ca2 + transmits information in a concentration oscillation manner). Various calcium channels related to Ca2 + influx, such as voltage-gated calcium channels, nonselective calcium channels, Na + -Ca2 + exchangers, and calcium pumps, can influence the Ca2 + concentration, thereby affecting the osteogenic function of mesenchymal stem cells.

Furthermore, the results of the transcription factor analysis suggest potential regulatory mechanisms. JUND, DDIT3, SOX4, and STAT1 were highly enriched in C0 MSCs (Fig. 6F). JunD, a transcription factor that belongs to the family of activating protein-1 (AP-1) transcription factors, along with SOX4, which is associated with the Epithelial-mesenchymal transition (EMT) pathway, may promote the expression of genes involved in bone morphogenesis. Additionally, the endoplasmic reticulum stress-related transcription factor DDIT3 potentially influences MSC differentiation by regulating downstream ITGA11 and DHRS3 in response to stimuli. In addition, PRRX1, STAT3, and FOXP1 were noted in the C1 cluster, with SMAD7 and GLI3 as regulons (Fig. 6F). FOXP1 deficiency is associated with premature bone loss and bone defects in mice, suggesting that FOXP1 may affect the Ca2 + concentration and osteogenesis. Moreover, osteogenic subunits exhibit genetic characteristics that vary during the progression of multiple myeloma (FS4D). We found that, compared with those in HD samples, RRAS2, SYNCRIP, GLO1, TWSG1, FERMT2, and TNC were upregulated in RISS-I samples, whereas HDAC7, CTSK, SUN1, RUNX1, SMAD5, CTNNB1, and ADAR were more highly expressed in R-ISS III samples (Fig. 6G). Among them, high expression of CTNNB1 is associated with better prognosis, and RUNX1 has been reported in previous work as a vital osteogenesis regulon in MM (Fig. 6H). Further studies are needed to confirm their precise contributions.

In conclusion, our results highlight the distinct roles of osteogenic functional C0 and C1 MSCs in MM, emphasizing the need for further investigations into targeted therapies that modulate MSC behavior to improve patient outcomes. Future studies should focus on the therapeutic potential of manipulating these specific MSC subpopulations to increase osteoblastic differentiation and inhibit tumor progression.

## Discussion

In this study, we explored the heterogeneity of MSCs in the bone marrow microenvironment of MM patients using single-cell RNA sequencing (scRNA-seq) and bulk transcriptomic data. Our findings demonstrate the pivotal role of MSC subpopulations in shaping disease progression and the immune landscape within the tumor microenvironment (TME), with implications for immune suppression, drug resistance, and bone damage.

A key observation in our study was the identification of distinct MSC subpopulations, including immune regulatory, osteogenic, angiogenic, and multipotent clusters, each contributing uniquely to the pathology of MM. Immune regulatory MSCs, in particular, appear to play a critical role in creating an immunosuppressive environment that facilitates tumor immune evasion. These MSCs likely promote immune suppression through the secretion of cytokines and direct interactions with immune cells, preventing effective antitumor responses. These findings align with earlier studies that emphasized the role of MSCs in inhibiting immune cell activation, particularly T-cell responses, which are crucial for combating malignancies. CD138 (SDC1), CD319 (SLAMF7), and BCMA (TNFRSF17) are targets for plasma cells in immunotherapy, and we propose similar candidates for a unique cluster of MSCs in MM. Our results suggest that targeting immune regulatory HMGhMSCs could be a novel therapeutic approach to enhance immune activation and improve treatment outcomes in MM.

Furthermore, the decrease in the number of osteogenic MSCs as the disease progresses from early to advanced R-ISS stages underscores the link between MSC function and the severe osteolysis observed in MM patients. MSCs are known to differentiate into osteoblasts, which are responsible for bone formation; however, as MM advances, this osteogenic potential diminishes, leading to increased bone resorption and lytic lesions. This observation is consistent with the clinical manifestations of MM, where bone disease remains a major cause of morbidity. Our data support the hypothesis that enhancing the osteogenic differentiation of MSCs could provide therapeutic benefits, potentially reducing bone damage and improving the quality of life of MM patients.

The prognostic model we developed, which is based on MSC gene expression profiles, significantly contributes to the field by offering a tool to predict patient outcomes. Our analysis revealed that higher levels of immunoregulatory MSCs are correlated with poorer overall survival, whereas an abundance of osteogenic MSCs is associated with better outcomes. These findings highlight the prognostic significance of MSCs and suggest that these cells could serve as both biomarkers for disease progression and therapeutic targets. By distinguishing high-risk patients from low-risk patients on the basis of MSC profiles, this model has the potential to guide more personalized treatment strategies, allowing for earlier intervention and targeted therapies to improve prognosis.

While our study advances the understanding of MSC heterogeneity in MM, there are several avenues that warrant further investigation. The precise molecular mechanisms that govern MSC differentiation and their interactions with other cell types in the TME remain unknown. It is also important to explore how MSCs interact with cancer-associated fibroblasts, myeloid cells, and lymphocytes in the bone marrow, which may reveal additional pathways that contribute to disease progression and resistance to therapy. Moreover, the potential of MSCs to transition into cancer-associated fibroblasts, as suggested by our data, offers another intriguing avenue for research, particularly in understanding their role in promoting tumor growth and metastasis.

Future studies should also focus on developing therapeutic interventions aimed at modulating specific MSC subpopulations. For example, enhancing the osteogenic potential of MSCs could help mitigate bone disease, while targeting immune regulatory MSCs could improve immune responses against tumors. Additionally, further clinical validation of our prognostic model in larger patient cohorts is necessary to confirm its utility and refine its predictive power.

In conclusion, our study provides a comprehensive analysis of the heterogeneity of MSCs in multiple myeloma and highlights their critical roles in immune suppression, disease progression, and bone pathology. By identifying distinct MSC subpopulations and linking them to prognosis, we lay the groundwork for future therapeutic strategies that target MSCs to improve outcomes in MM patients. Our findings underscore the need for continued research into the dynamic interactions between MSCs and the TME, which could unlock new avenues for treating this complex and challenging disease.

## Conclusion

This study elucidates the critical role of mesenchymal stem cell (MSC) heterogeneity in shaping the tumor microenvironment of multiple myeloma (MM), revealing distinct functional subpopulations that drive disease progression and therapeutic resistance. Through integrated single-cell RNA sequencing and bulk transcriptomic analyses, we identified four MSC clusters—osteogenic, angiogenic, immune regulatory, and multipotent—each contributing uniquely to MM pathogenesis. Notably, the osteogenic MSC population, essential for bone homeostasis, diminishes with advancing R-ISS stages, correlating with progressive bone destruction in late-stage disease. Concurrently, immune regulatory MSCs foster an immunosuppressive niche, facilitating tumor immune evasion. A novel subclone, HMGhMSC, characterized by upregulated high mobility group protein expression, emerged as a potent regulator within the microenvironment, influencing tumor-stroma crosstalk and drug resistance. Leveraging these insights, we developed a prognostic model centered on HMGhMSC dynamics, demonstrating its potential to predict clinical outcomes and guide therapeutic strategies. Targeting HMGhMSC or restoring osteogenic MSC function may counteract bone lysis and immunosuppression, offering dual benefits for MM treatment. These findings underscore the therapeutic relevance of MSC subpopulation modulation, advocating for precision approaches to disrupt microenvironmental support mechanisms and improve patient prognosis. These targets can be validated in further preclinical models and explore combinatorial therapies to enhance efficacy in MM management.

While our findings provide novel insights, several limitations should be acknowledged. First, the restricted sample size of our single-cell cohort may affect the generalizability of identified MSC subpopulations. Second, although we characterized HMGhMSC's functional properties through bioinformatic analyses, experimental validation of key mechanisms (particularly HMGB1-mediated signaling pathways) remains necessary. Third, our prognostic model requires multicenter validation in larger, independent cohorts to confirm its clinical utility across diverse patient populations. Future studies should: (1) exploring the possible mechanisms by which HMGhMSC regulates the bone marrow microenvironment of multiple myeloma, (2) investigate MSC-carcinoma cell interactions using advanced 3D coculture systems, and evaluate combination therapies targeting both MSC subpopulations and malignant plasma cells, (3) prospective validation in multi-institutional cohorts to demonstrate clinical translatability of novel prognostic model.

## Supplementary Information

Additional file 1: Fig. S1. Single-cell transcriptomic analysis of MSC heterogeneity in multiple myelomaprogression and healthy donor.Quality control.Cell annotation.The numbers of major cell type in multiple myeloma progression and HD.The number of differentially expressed genesamong major cell types in MM.Heatmap showing the five most variable genes across functional major cells. Fig. S2. Single-cell landscape of iregMSCs in multiple myeloma at different stages.Heatmap representing the signature matrix generated by CIBERSORTx based on the current single-cell sequencing data, used for deconvolution of bulk sequencing:major cell types andsubpopulations.Heatmap showing the five most variable genes across subpopulations of iregMSCscells.The numbers of subpopulations of iregMSCs cells in multiple myeloma progression and HD.Heatmap showing the expression of prognostic DEGs in iregMSC subpopulations during MM progression and HD.Pseudotime trajectories of stemness MSCs and iregMSC subpopulations inferred using the Slingshot algorithm. Fig. S3. Establishment and Evaluation of a Prognostic Model Based on the HMGhMSC. A KM curves of the 20 key genes in the prognostic model.LASSO regression coefficient of key prognostic genes.Distribution of prognostic gene expression scores, survival status, and heatmap of multiple myeloma patients under the risk scoring system.The performance of the model in the TT3-treated cohort from the GSE24080 dataset. Overall survivalanalysis. Event-free survivalanalysis.Comparison between the proposed prognostic model and ISS. C-index analysis. Time-dependent ROC curves at 1-, 3-, and 5-year survival. Fig. S4. Single-cell landscape of osteogenic MSCs in multiple myeloma at different stages. A The percentage of osteogenic MSC subpopulations in multiple myeloma progression and HD.Heatmap showing the five most variable genes across osteogenic MSC subpopulations.The number of differentially expressed genesamong osteogenic MSC subpopulations in MM.Heatmap showing the expression of prognostic DEGs in osteogenic MSC subpopulations during MM progression and HD

## Data Availability

The datasets used and/or analysed during the current study are available from the corresponding author on reasonable request.

## Abbreviations

MM: Multiple myeloma
MSCs: Mesenchymal stem cells
R-ISS: Revised International Staging System
RRMM: Relapsed and refractory multiple myeloma
BME: Bone marrow environment
Tregs: Regulatory T cells
MDSCs: Myeloid-derived suppressor cells
MGUS: Monoclonal gammopathy of undetermined significance
SMM: Smoldering multiple myeloma
CAFs: Cancer-associated fibroblasts
TLR4: Toll-like receptor 4
IL-6: Interleukin 6
scRNA-seq: Single-cell RNA sequencing
NDMM: Newly diagnosed multiple myeloma
UMI: Unique molecular identifier
ROC: Receiver operating characteristic
HR: Hazard ratios
CI: Confidence intervals
IL-8: Interleukin-8
BM: Bone marrow
TME: Tumor microenvironment
EMT: Epithelial‒mesenchymal transition
HMGhMSCs: High mobility group proteins highly up-expressed mesenchymal stem cells
TFs: Transcription factors
CXCL12: C-X-C motif chemokine ligand 12
NK cells: Natural killer cell
DCs: Dendritic cells
DEGs: Differentially expressed genes
EMT: Epithelial-to-mesenchymal transition
AP-1: Activating protein-1
JAK-STAT: Janus kinase-signal transducer and activator of transcription
FDR: False discovery rate
NF-κB: Nuclear Factor-Kappa B
MAPK: Mitogen-activated protein kinase
