# Supplementary figures and images for: Integrative analysis of bulk and single-cell gene expression profiles to identify bone marrow mesenchymal cell heterogeneity and prognostic significance in multiple myeloma
Source: J Transl Med. 2025 Jun 16;23:659. doi: 10.1186/s12967-025-06637-6 (PMC12172380; doi:10.1186/s12967-025-06637-6)

A

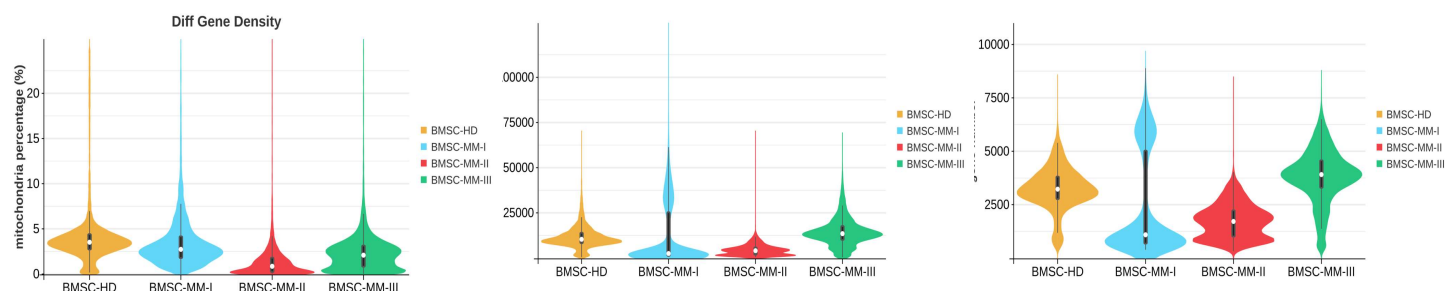

B

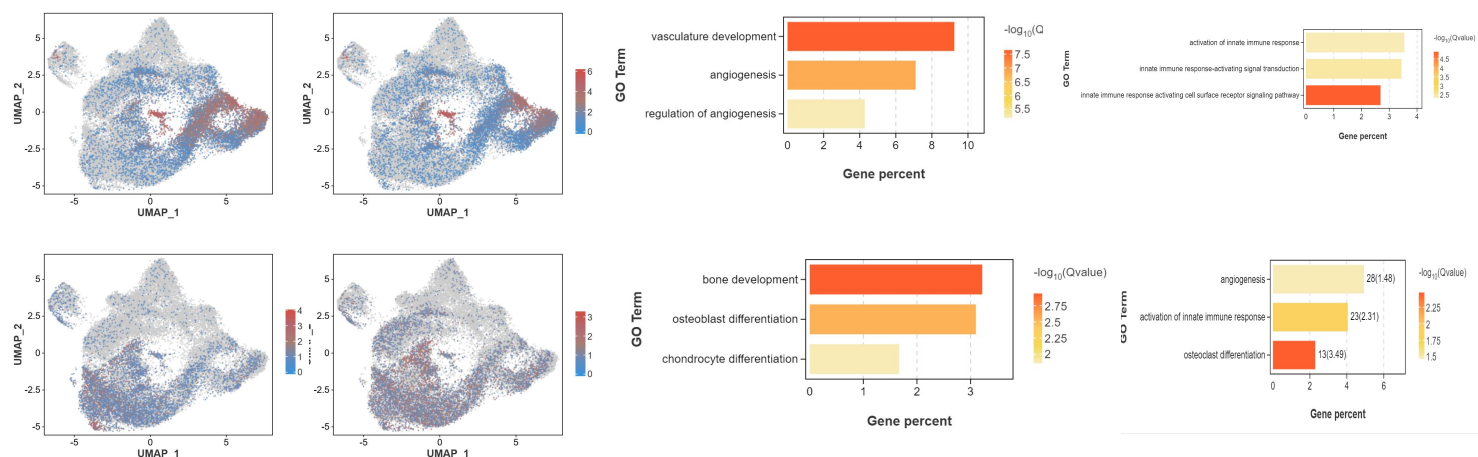

C

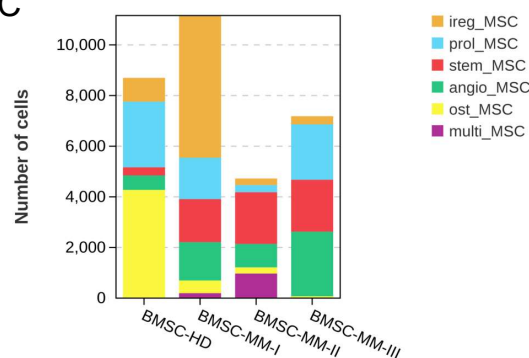

D

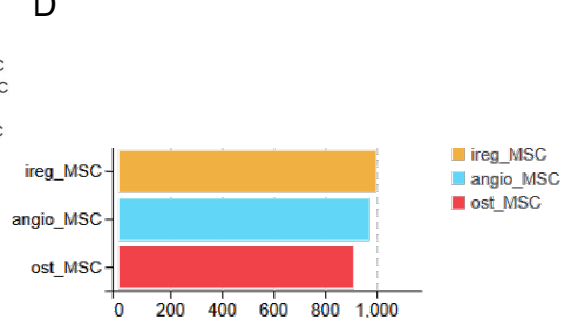

E

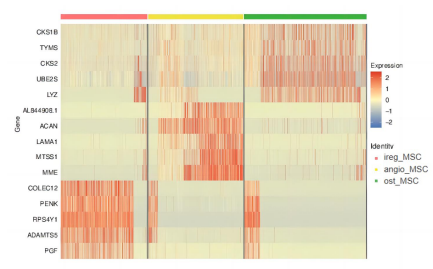

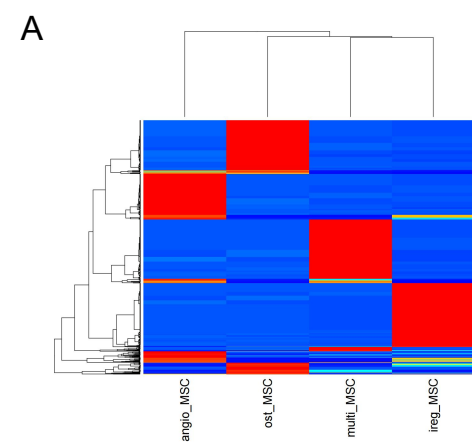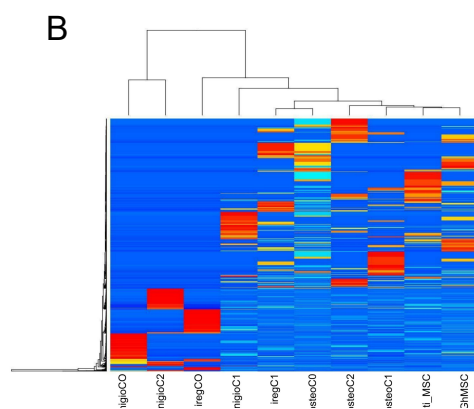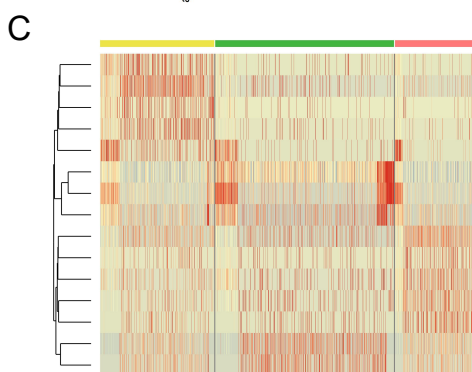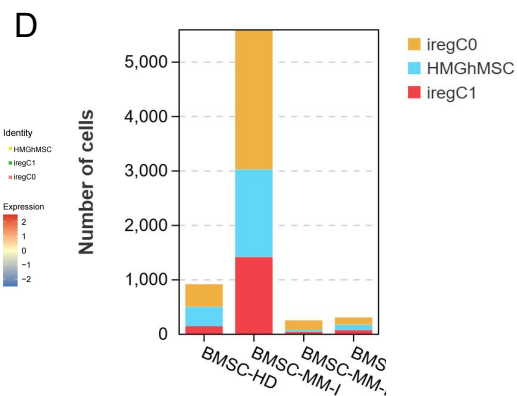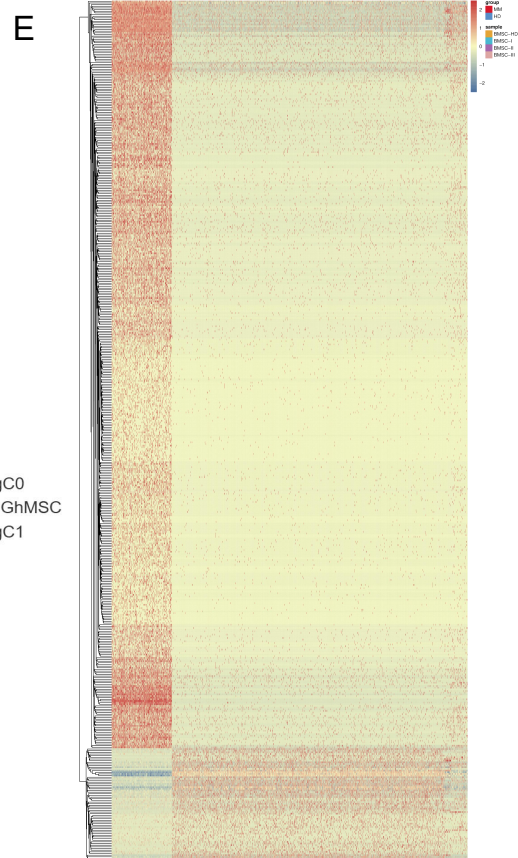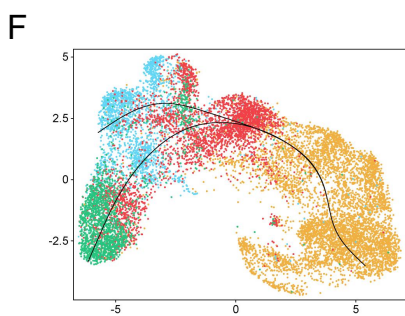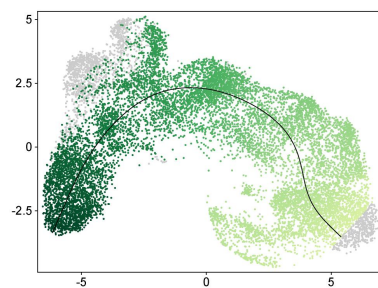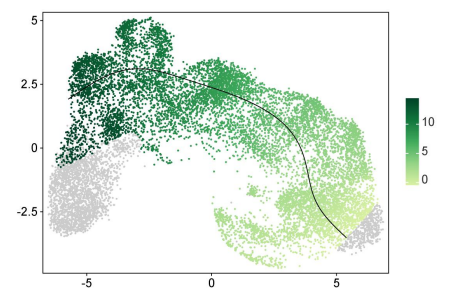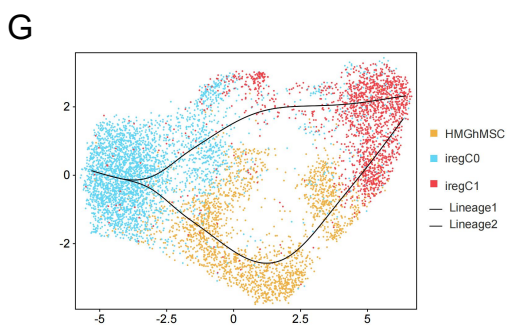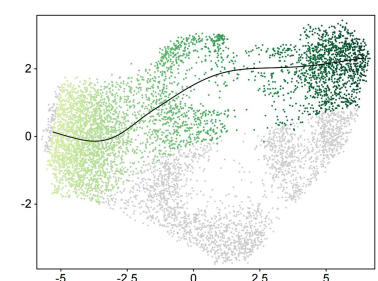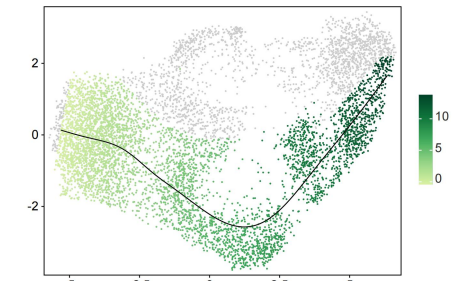

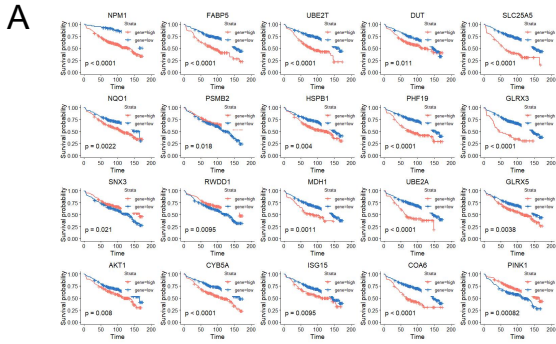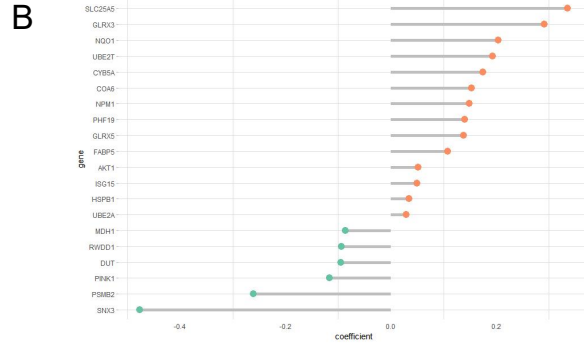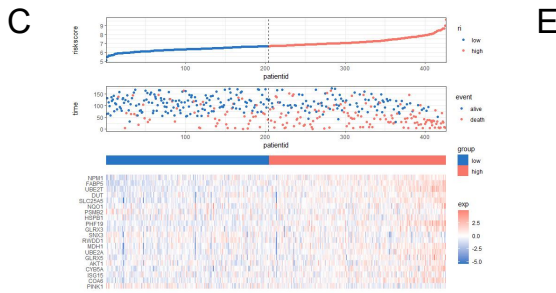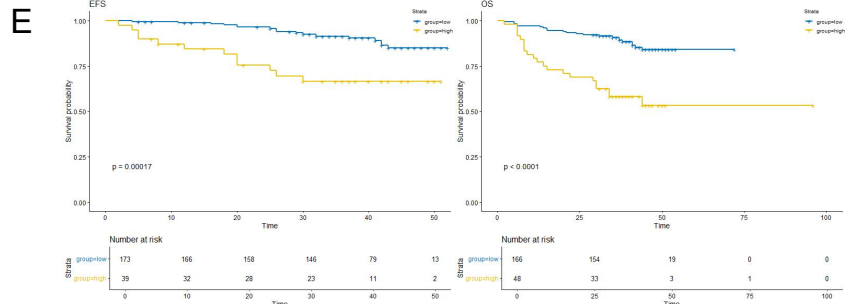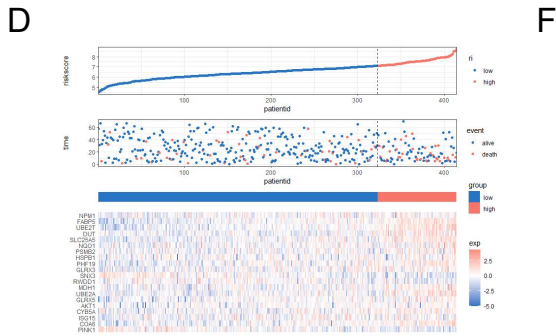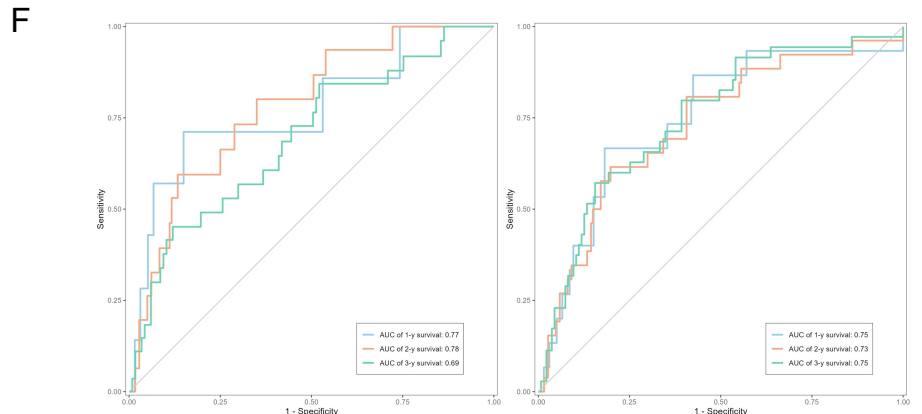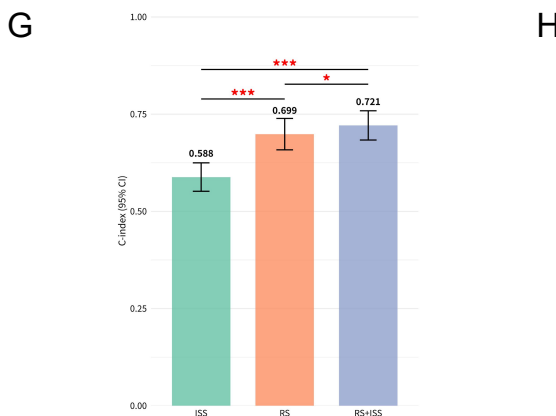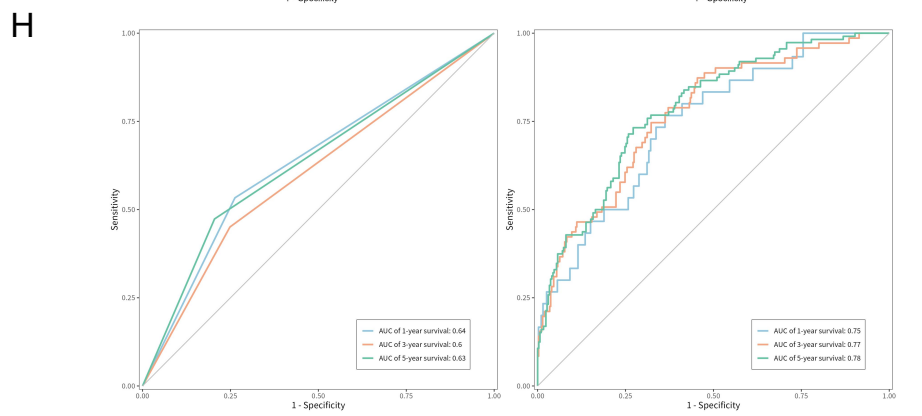

A

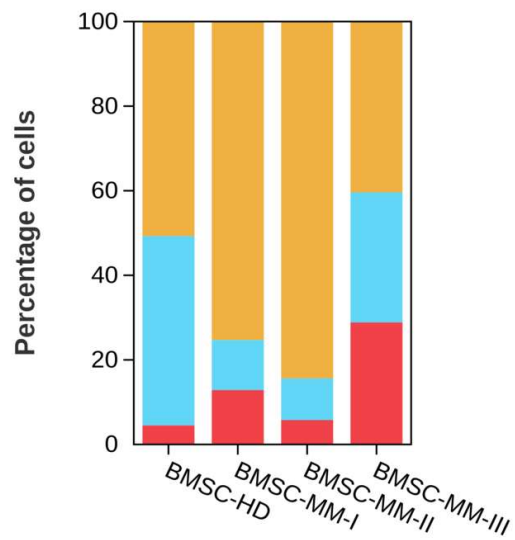

B

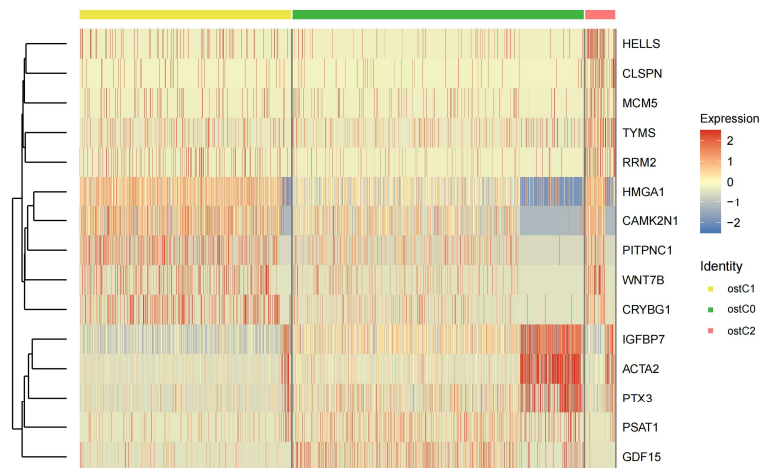

C

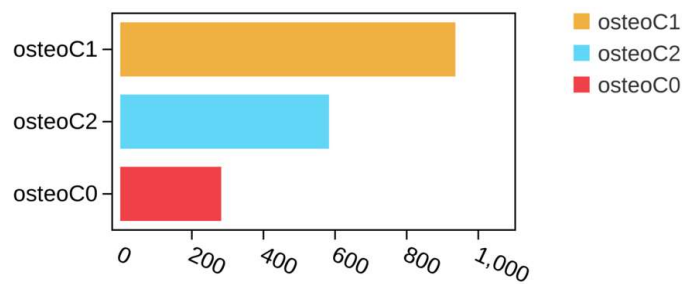

D

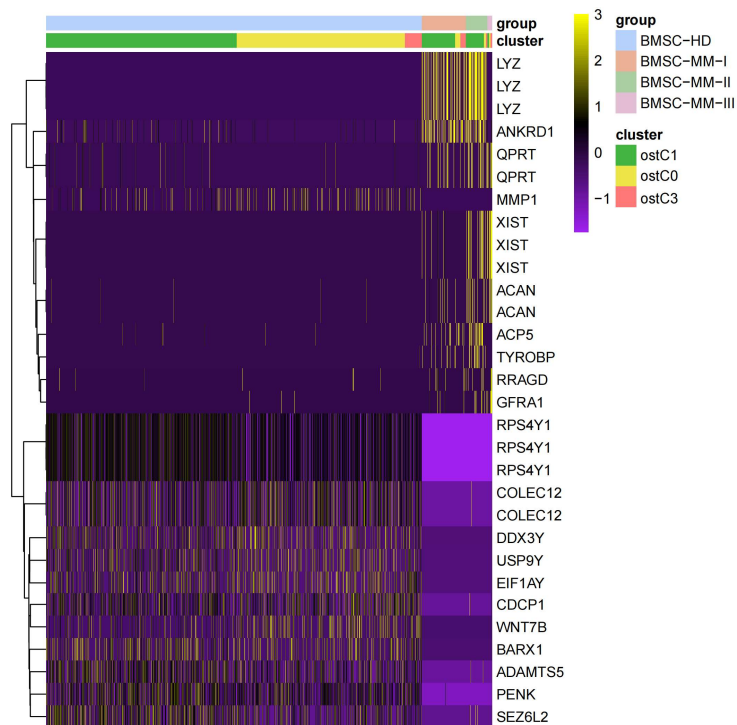

Supplement: Supplementary file 1 — Additional file 1: Fig. S1. Single-cell transcriptomic analysis of MSC heterogeneity in multiple myelomaprogression and healthy donor.Quality control.Cell annotation.The numbers of major cell type in multiple myeloma progression and HD.The number of differentially expressed genesamong major cell types in MM.Heatmap showing the five most variable genes across functional major cells. Fig. S2. Single-cell landscape of iregMSCs in multiple myeloma at different stages.Heatmap representing the signature matrix generated by CIBERSORTx based on the current single-cell sequencing data, used for deconvolution of bulk sequencing:major cell types andsubpopulations.Heatmap showing the five most variable genes across subpopulations of iregMSCscells.The numbers of subpopulations of iregMSCs cells in multiple myeloma progression and HD.Heatmap showing the expression of prognostic DEGs in iregMSC subpopulations during MM progression and HD.Pseudotime trajectories of stemness MSCs and iregMSC subpopulations inferred using the Slingshot algorithm. Fig. S3. Establishment and Evaluation of a Prognostic Model Based on the HMGhMSC. A KM curves of the 20 key genes in the prognostic model.LASSO regression coefficient of key prognostic genes.Distribution of prognostic gene expression scores, survival status, and heatmap of multiple myeloma patients under the risk scoring system.The performance of the model in the TT3-treated cohort from the GSE24080 dataset. Overall survivalanalysis. Event-free survivalanalysis.Comparison between the proposed prognostic model and ISS. C-index analysis. Time-dependent ROC curves at 1-, 3-, and 5-year survival. Fig. S4. Single-cell landscape of osteogenic MSCs in multiple myeloma at different stages. A The percentage of osteogenic MSC subpopulations in multiple myeloma progression and HD.Heatmap showing the five most variable genes across osteogenic MSC subpopulations.The number of differentially expressed genesamong osteogenic MSC subpopulations in MM.Heatmap [file 12967_2025_6637_MOESM1_ESM.pdf]
